# Tetra­kis(tri­ethyl­enediamin-1-ium) dodeca-μ_2_-chlorido-hexakis(thio­cyanato-κ*N*)hexa-*octa­hedro*-niobate

**DOI:** 10.1107/S241431462300398X

**Published:** 2023-05-12

**Authors:** Eric Sperlich, Martin Köckerling

**Affiliations:** a Universität Potsdam, Institut für Chemie, Liebknecht-Str. 24-25, D-14476 Potsdam, Germany; b Universität Rostock, Institut für Chemie, Anorganische Festkörperchemie, Albert-Einstein-Str. 3a, D-18059 Rostock, Germany; Vienna University of Technology, Austria

**Keywords:** crystal structure, niobium, cluster, chloride, DABCO

## Abstract

The crystal structure of the cluster complex salt, (C_6_H_13_N_2_)_4_[Nb_6_(NCS)_6_Cl_12_] or (H-DABCO)_4_[Nb_6_Cl_12_(NCS)_6_] (DABCO = tri­ethyl­enedi­amine or 1,4-di­aza­bicyclo­[2.2.2]octa­ne), comprises octa­hedral Nb_6_ cluster cores, which are μ_2_-coordinated by 12 chloride ligands (bridging the octa­hedral edges, *inner* ligand sphere). Furthermore, each Nb atom is N-bonded to a terminal thio­cyanate ligand (*outer* ligand sphere). The discrete clusters carry a charge of −4, which is compensated by four monoprotonated DABCO mol­ecules. These are arranged in rows, which are N—H⋯Cl and N—H⋯N hydrogen bonded to the anions and among each other.

## Abstract 2

The anion of the cluster complex salt, (H-DABCO)_4_[Nb_6_Cl_12_(NCS)_6_], comprises octa­hedral Nb_6_ cluster units, which are coordinated by twelve *μ*_2_-bridging chlorido ligands and six terminal N-bound thio­cyanato ligands.

## Graphical abstract

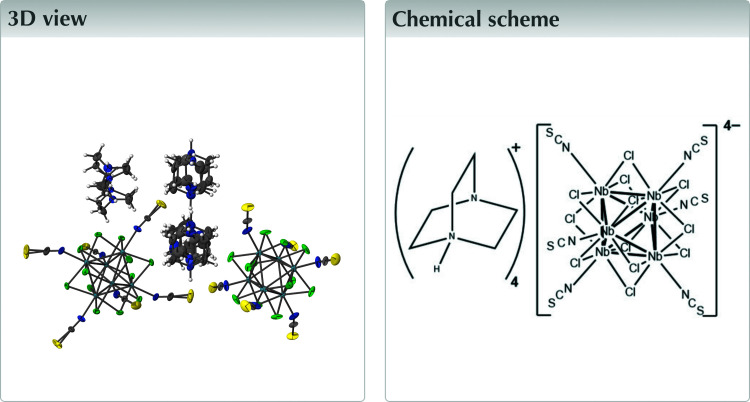

## Structure description

**Figure 1:**
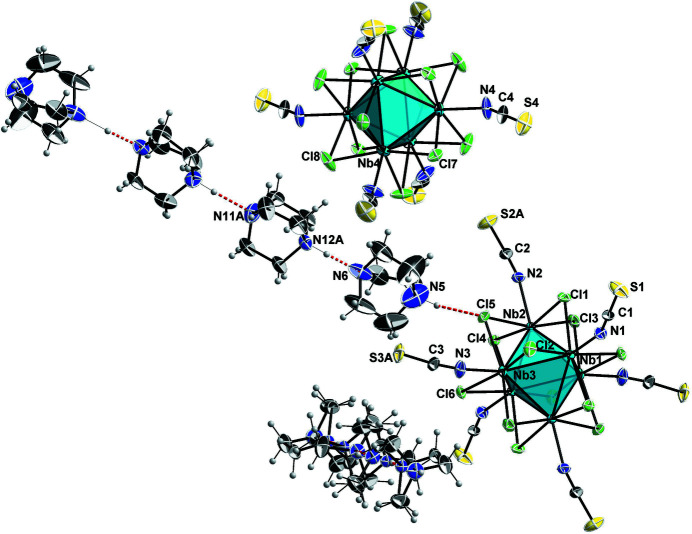
The structures of the discrete anionic cluster units and rows of cation units of (H-DABCO)_4_[Nb_6_Cl_12_(NCS)_6_]. Atoms are drawn with displacement ellipsoids at the 50% probability level. The Nb_6_ metal atom octa­hedron is shown in a polyhedral representation, and N—H⋯N and N—H⋯Cl hydrogen bonds as red dashed lines. Of the disordered parts, only one of each is displayed for better visibility.

**Figure 2:**
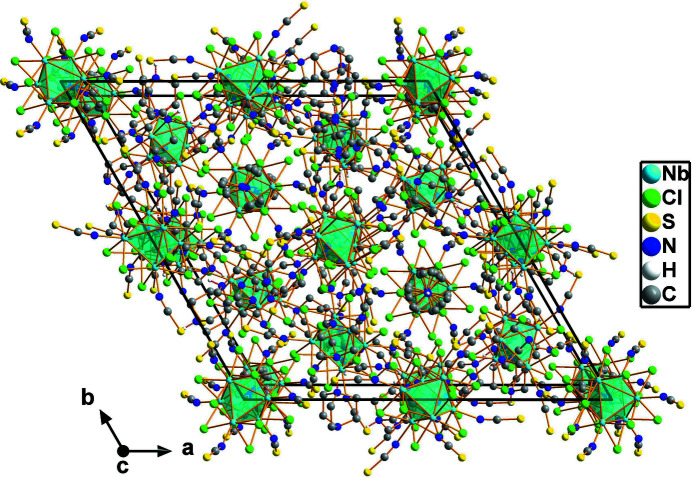
Arrangement of cluster anions and (H-DABCO) cations in the unit cell in a view along the crystallographic *c* axis. The Nb_6_ metal atom octa­hedra are shown in a polyhedral representation.

Cluster complexes with octa­hedral cores of electron-poor transition metals, exhibiting strong metal–metal bonds, have been investigated for many decades (Braunstein *et al.*, 1999 ▸; Cotton, 1964 ▸; Dehnen, 2017 ▸; Janiak *et al.*, 2012 ▸; Simon, 1988 ▸; Vaughan *et al.*, 1950 ▸). Starting from [Nb_6_Cl_12_(CH_3_OH)_4_(OCH_3_)_2_]·DABCO·0.66CH_2_Cl_2_ (Sperlich & Köckerling, 2021 ▸), the title compound was obtained by ligand-exchange reactions with thio­cyanate salts in methanol. The crystal structure of the title compound consists of discrete [Nb_6_Cl_12_(NCS)_6_]^4−^ cluster anions and (H-DABCO)^+^ cations. The Nb atoms of the cluster anions are arranged octa­hedrally. The octa­hedral edges are *μ*_2_-bridged by Cl^−^ ligands and the *exo* sites occupied by the N-binding NCS^−^ ligands. Two symmetry-independent cluster units are present in the unit cell. One unit is located at the Wyckoff site 9*e* with point-group symmetry 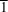 , the other at site 3*a* with point-group symmetry 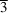 . of the space group *R* 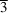 . The cations are arranged in N—H⋯Cl and N—H⋯N hydrogen-bonded rows of four protonated DABCO mol­ecules between two cluster units. Two H-DABCO cations (comprising atoms N7, N8, and N9, N10) are situated on a threefold rotation axis, and one H-DABCO cation (comprising N11, N12) is statistically disordered over two sets of sites. The four protons per cationic row are statistically attached to the five possible sites. Selected hydrogen bonds are listed in Table 1 ▸. The resulting structural arrangement is shown in Fig. 1 ▸. The inter­atomic distances in the individual cations and anions are found in the expected regions. For both cluster units they are in the range of Nb_6_ cluster compounds with 16 cluster-based electrons, in line with the charge of −4. Comparable *A*_4_[Nb_6_Cl_12_(NCS)_6_] salts with the same discrete cluster anion have been reported for *A* = K, Rb, and NH_4_ (Reckeweg & Meyer, 1996 ▸), Ph_4_P (Flemming *et al.*, 2009 ▸), and Cs (Naumov *et al.*, 2003 ▸). Fig. 2 ▸ shows the hexa­gonal packing of the cluster units with inter­mediate H-DABCO cations. Starting from compounds with such discrete iso-thio­cyanato ligated cluster units, cluster network compounds have already been synthesized (Pigorsch & Köckerling, 2016 ▸).

## Synthesis and crystallization

**Figure 3:**
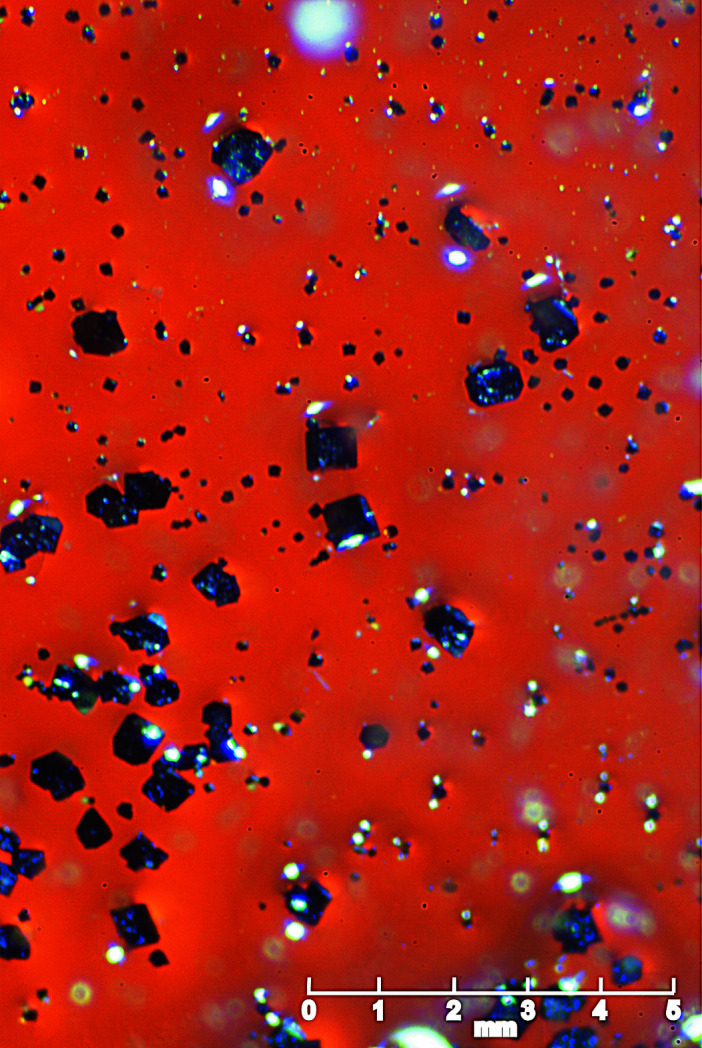
Microscopic view of crystals of (H-DABCO)_4_[Nb_6_Cl_12_(NCS)_6_].

The cluster compound [Nb_6_Cl_12_(CH_3_OH)_4_(OCH_3_)_2_]·DABCO·0.66CH_2_Cl_2_ was used as starting material (Sperlich & Köckerling, 2021 ▸). In a glass vial of 4 ml volume, 20 mg (15.56 µmol) of the precursor, 9 mg (93.37 µmol) of potassium thio­cyanate, KSCN, 12 mg (157.64 µmol) of ammonium thio­cyanate, (NH_4_)SCN, and 1 ml of methanol were filled. The vial was placed in a sand bath at 313 K. After one day it was taken out of the sand bath and allowed to stand untouched for several days at room temperature. During this time, the title compound crystallized from the reaction mixture in the form of black crystals (Fig. 3 ▸). For analytically pure samples, the crystals were filtered from the solution and were washed with anhydrous ethanol and anhydrous methyl­ene chloride. Yields were up to 80%.

## Refinement

Crystal data, data collection and structure refinement details are summarized in Table 2 ▸. The sulfur atoms of two thio­cyanato groups (S2 and S3) are disordered over two sets of sites, both in a ratio of 0.51 (3):0.49 (3). One of the four H-DABCO units (comprising atoms N11 and N12) is equally disordered over two sets of sites, denoted as *A* and *B*.

## Supplementary Material

Crystal structure: contains datablock(s) I. DOI: 10.1107/S241431462300398X/wm4186sup1.cif

Structure factors: contains datablock(s) I. DOI: 10.1107/S241431462300398X/wm4186Isup2.hkl

CCDC reference: 2260596

Additional supporting information:  crystallographic information; 3D view; checkCIF report

## Figures and Tables

**Table 1:** Hydrogen-bond geometry (Å, °)

*D*—H⋯*A*	*D*—H	H⋯*A*	*D*⋯*A*	*D*—H⋯*A*
N5—H5*N*⋯Cl5	1.00	2.46	3.368 (7)	150
N8—H8*N*⋯N9	1.00	1.80	2.795 (8)	180
N10—H10*N*⋯Cl7^i^	1.00	2.90	3.695 (5)	137
N12A—H12N⋯N6^ii^	1.00	1.59	2.59 (1)	176

Symmetry codes: (i) 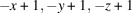 ; (ii) 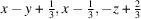 .

**Table 2:** Experimental details

Crystal data	
Chemical formula	(C_6_H_13_N_2_)_4_[Nb_6_(NCS)_6_Cl_12_]
*M*_r_	1784.08
Crystal system, space group	Trigonal, *R* 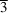
Temperature (K)	123
*a*, *c* (Å)	25.399 (2), 30.418 (2)
*V* (Å^3^)	16993 (3)
*Z*	12
Radiation type	Mo *K*α
μ (mm^−1^)	1.98
Crystal size (mm)	0.30 × 0.20 × 0.20

Data collection	
Diffractometer	Bruker APEXII CCD
Absorption correction	Multi-scan (*SADABS*; Krause *et al.*, 2015 ▸)
No. of measured, independent and observed [*I* > 2σ(*I*)] reflections	84629, 13784, 11834
*R*_int_	0.040
(sin θ/λ)_max_ (Å^−1^)	0.758

Refinement	
*R*[*F* ^2^ > 2σ(*F* ^2^)], *wR*(*F* ^2^), *S*	0.040, 0.093, 1.09
No. of reflections	13784
No. of parameters	502
H-atom treatment	H atoms treated by a mixture of independent and constrained refinement
Δρ_max_, Δρ_min_ (e Å^−3^)	2.03, −1.79

Computer programs: *APEX3* and *SAINT* (Bruker, 2017 ▸), *SHELXS* (Sheldrick, 2008 ▸), *SHELXL* (Sheldrick, 2015 ▸), *DIAMOND* (Brandenburg & Putz, 2019 ▸) and *publCIF* (Westrip, 2010 ▸).

## full crystallographic data

### Crystal data

**Table:**

(C_6_H_13_N_2_)_4_[Nb_6_(NCS)_6_Cl_12_]	*D*_x_ = 2.092 Mg m^−^^3^
*M**_r_* = 1784.08	Mo *K*α radiation, λ = 0.71073 Å
Trigonal, *R*3	Cell parameters from 9867 reflections
*a* = 25.399 (2) Å	θ = 2.5–32.6°
*c* = 30.418 (2) Å	µ = 1.98 mm^−^^1^
*V* = 16993 (3) Å^3^	*T* = 123 K
*Z* = 12	Block, black
*F*(000) = 10512	0.30 × 0.20 × 0.20 mm

### Data collection

**Table:**

Bruker APEXII CCD diffractometer	11834 reflections with *I* > 2σ(*I*)
Radiation source: microfocus sealed tube	*R*_int_ = 0.040
φ and ω scans	θ_max_ = 32.6°, θ_min_ = 1.1°
Absorption correction: multi-scan (SADABS; Krause *et al.*, 2015)	*h* = −38→38
	*k* = −38→38
84629 measured reflections	*l* = −46→46
13784 independent reflections	

### Refinement

**Table:**

Refinement on *F*^2^	Primary atom site location: structure-invariant direct methods
Least-squares matrix: full	Secondary atom site location: difference Fourier map
*R*[*F*^2^ > 2σ(*F*^2^)] = 0.040	Hydrogen site location: inferred from neighbouring sites
*wR*(*F*^2^) = 0.093	H atoms treated by a mixture of independent and constrained refinement
*S* = 1.09	*w* = 1/[σ^2^(*F*_o_^2^) + (0.020*P*)^2^ + 223.5098*P*] where *P* = (*F*_o_^2^ + 2*F*_c_^2^)/3
13784 reflections	(Δ/σ)_max_ = 0.002
502 parameters	Δρ_max_ = 2.03 e Å^−^^3^
0 restraints	Δρ_min_ = −1.79 e Å^−^^3^

### Special details

**Table:**

Geometry. All esds (except the esd in the dihedral angle between two l.s. planes) are estimated using the full covariance matrix. The cell esds are taken into account individually in the estimation of esds in distances, angles and torsion angles; correlations between esds in cell parameters are only used when they are defined by crystal symmetry. An approximate (isotropic) treatment of cell esds is used for estimating esds involving l.s. planes.
Refinement. C-and N-bound hydrogen atoms were placed on idealized positions and refined using a riding models.

### Fractional atomic coordinates and isotropic or equivalent isotropic displacement parameters (Å^2^)

**Table:**

	*x*	*y*	*z*	*U*_iso_*/*U*_eq_	Occ. (<1)
Nb1	0.15274 (2)	0.27928 (2)	0.38766 (2)	0.01794 (5)	
Nb2	0.21875 (2)	0.30094 (2)	0.30483 (2)	0.01810 (5)	
Nb3	0.24363 (2)	0.40316 (2)	0.36253 (2)	0.01897 (5)	
Nb4	0.65963 (2)	0.39594 (2)	0.37251 (2)	0.02176 (6)	
Cl1	0.20921 (4)	0.22977 (4)	0.36270 (3)	0.0306 (2)	
Cl2	0.24134 (4)	0.35140 (4)	0.43131 (3)	0.0304 (2)	
Cl3	0.13796 (4)	0.21431 (3)	0.26443 (3)	0.0296 (2)	
Cl4	0.24550 (4)	0.35960 (4)	0.23630 (2)	0.0260 (1)	
Cl5	0.31827 (3)	0.37626 (4)	0.33539 (3)	0.0296 (2)	
Cl6	0.27439 (3)	0.47858 (3)	0.30297 (3)	0.0271 (1)	
Cl7	0.73993 (4)	0.41477 (4)	0.42584 (2)	0.0305 (2)	
Cl8	0.72998 (6)	0.48879 (4)	0.33235 (3)	0.0453 (3)	
S1	0.17220 (6)	0.16375 (5)	0.50800 (4)	0.0499 (3)	
S2B_a	0.3823 (2)	0.2647 (2)	0.2775 (6)	0.065 (2)	0.49 (3)
S2A_b	0.3887 (3)	0.2711 (4)	0.2966 (3)	0.049 (2)	0.51 (3)
S3A_c	0.4484 (1)	0.5661 (4)	0.3962 (4)	0.043 (1)	0.49 (3)
S3B_d	0.4442 (2)	0.5774 (2)	0.3810 (3)	0.035 (1)	0.51 (3)
S4	0.67419 (8)	0.51041 (6)	0.50030 (4)	0.0591 (4)	
N1	0.1412 (1)	0.2221 (1)	0.4453 (1)	0.0304 (6)	
N2	0.2775 (2)	0.2684 (1)	0.2764 (1)	0.0343 (7)	
N3	0.3273 (1)	0.4780 (2)	0.3911 (1)	0.0355 (7)	
N4	0.6530 (2)	0.4609 (2)	0.4167 (1)	0.0454 (9)	
N5	0.4310 (4)	0.4556 (4)	0.2629 (2)	0.102 (2)	
H5N	0.3886	0.4307	0.2745	0.123*	
N6	0.5372 (3)	0.5180 (2)	0.2347 (1)	0.062 (1)	
N7	0.3333	0.6667	0.2131 (2)	0.0427 (13)	
N8	0.3333	0.6667	0.2968 (2)	0.0291 (10)	
H8N	0.3333	0.6667	0.3297	0.035*	
N9	0.3333	0.6667	0.3887 (2)	0.0270 (9)	
N10	0.3333	0.6667	0.4714 (2)	0.0450 (14)	
H10N	0.3333	0.6667	0.5043	0.054*	
C1	0.1537 (2)	0.1978 (2)	0.4716 (1)	0.0248 (6)	
C2	0.3228 (2)	0.2677 (2)	0.2819 (2)	0.045 (1)	
C3	0.3772 (2)	0.5172 (2)	0.3901 (1)	0.0341 (7)	
C4	0.6618 (2)	0.4819 (2)	0.4516 (1)	0.0365 (8)	
C5	0.4472 (5)	0.5205 (5)	0.2635 (3)	0.126 (4)	
H5A	0.4258	0.5292	0.2399	0.151*	
H5B	0.4370	0.5316	0.2922	0.151*	
C6	0.5137 (5)	0.5538 (4)	0.2561 (3)	0.111 (4)	
H6A	0.5243	0.5900	0.2378	0.133*	
H6B	0.5344	0.5684	0.2848	0.133*	
C7	0.4319 (3)	0.4367 (3)	0.2174 (2)	0.066 (2)	
H7A	0.3981	0.4355	0.2003	0.079*	
H7B	0.4277	0.3958	0.2167	0.079*	
C8	0.4923 (3)	0.4833 (3)	0.1985 (2)	0.068 (2)	
H8A	0.4867	0.5121	0.1798	0.082*	
H8B	0.5085	0.4628	0.1798	0.082*	
C9	0.4715 (4)	0.4440 (6)	0.2907 (3)	0.139 (5)	
H9A	0.4543	0.3998	0.2953	0.166*	
H9B	0.4772	0.4638	0.3197	0.166*	
C10	0.5314 (4)	0.4705 (3)	0.2665 (2)	0.080 (2)	
H10A	0.5654	0.4887	0.2879	0.097*	
H10B	0.5338	0.4378	0.2505	0.097*	
C11	0.3838 (3)	0.6592 (3)	0.2300 (2)	0.062 (1)	
H11A	0.4232	0.6961	0.2235	0.074*	
H11B	0.3834	0.6242	0.2154	0.074*	
C12	0.3769 (2)	0.6486 (2)	0.2798 (2)	0.0367 (8)	
H12A	0.3618	0.6051	0.2863	0.044*	
H12B	0.4169	0.6729	0.2944	0.044*	
C13	0.3955 (2)	0.6873 (2)	0.4054 (1)	0.0384 (8)	
H13A	0.4221	0.7312	0.3992	0.046*	
H13B	0.4128	0.6647	0.3904	0.046*	
C14	0.3926 (2)	0.6760 (3)	0.4550 (2)	0.050 (1)	
H14A	0.3961	0.6396	0.4612	0.060*	
H14B	0.4265	0.7114	0.4699	0.060*	
N11A_e	0.4625 (7)	0.4273 (5)	0.4797 (5)	0.057 (3)	0.5
N12A_e	0.4077 (5)	0.3171 (4)	0.4549 (4)	0.035 (2)	0.5
H12N_e	0.3860	0.2738	0.4449	0.042*	0.5
C15A_e	0.4859 (5)	0.3932 (5)	0.5062 (4)	0.054 (2)	0.5
H15A_e	0.4796	0.3967	0.5379	0.065*	0.5
H15B_e	0.5299	0.4104	0.5009	0.065*	0.5
C16A_e	0.4508 (4)	0.3260 (5)	0.4919 (3)	0.045 (2)	0.5
H16A_e	0.4800	0.3132	0.4823	0.054*	0.5
H16B_e	0.4277	0.3004	0.5173	0.054*	0.5
C17A_e	0.4761 (7)	0.4240 (5)	0.4331 (3)	0.065 (3)	0.5
H17A_e	0.5206	0.4429	0.4290	0.078*	0.5
H17B_e	0.4617	0.4468	0.4151	0.078*	0.5
C18A_e	0.4454 (5)	0.3588 (5)	0.4180 (3)	0.055 (3)	0.5
H18A_e	0.4764	0.3485	0.4086	0.066*	0.5
H18B_e	0.4189	0.3533	0.3925	0.066*	0.5
C19A_e	0.3974 (5)	0.4013 (6)	0.4859 (4)	0.060 (3)	0.5
H19A_e	0.3831	0.4251	0.4691	0.072*	0.5
H19B_e	0.3885	0.4030	0.5174	0.072*	0.5
C20A_e	0.3641 (5)	0.3353 (5)	0.4703 (4)	0.052 (2)	0.5
H20A_e	0.3397	0.3084	0.4947	0.062*	0.5
H20B_e	0.3361	0.3306	0.4460	0.062*	0.5
N11B_f	0.5240 (5)	0.5477 (5)	0.5162 (4)	0.047 (2)	0.5
H11N_f	0.5007	0.5040	0.5071	0.08 (4)*	0.5
N12B_f	0.5836 (6)	0.6587 (5)	0.5401 (4)	0.046 (2)	0.5
C15B_f	0.5711 (5)	0.5853 (5)	0.4820 (3)	0.053 (2)	0.5
H15C_f	0.5508	0.5875	0.4548	0.064*	0.5
H15D_f	0.5959	0.5664	0.4747	0.064*	0.5
C16B_f	0.6117 (6)	0.6492 (6)	0.5005 (4)	0.066 (3)	0.5
H16C_f	0.6520	0.6546	0.5082	0.079*	0.5
H16D_f	0.6178	0.6799	0.4780	0.079*	0.5
C17B_f	0.4833 (5)	0.5719 (6)	0.5220 (4)	0.065 (3)	0.5
H17C_f	0.4556	0.5515	0.5471	0.078*	0.5
H17D_f	0.4583	0.5644	0.4953	0.078*	0.5
C18B_f	0.5214 (6)	0.6400 (6)	0.5307 (6)	0.078 (4)	0.5
H18C_f	0.5196	0.6624	0.5046	0.094*	0.5
H18D_f	0.5039	0.6507	0.5559	0.094*	0.5
C19B_f	0.5587 (6)	0.5548 (6)	0.5587 (3)	0.064 (3)	0.5
H19C_f	0.5310	0.5260	0.5811	0.077*	0.5
H19D_f	0.5917	0.5456	0.5533	0.077*	0.5
C20B_f	0.5852 (7)	0.6194 (7)	0.5751 (4)	0.071 (3)	0.5
H20C_f	0.5617	0.6201	0.6008	0.085*	0.5
H20D_f	0.6278	0.6349	0.5846	0.085*	0.5

### Atomic displacement parameters (Å^2^)

**Table:**

	*U*^11^	*U*^22^	*U*^33^	*U*^12^	*U*^13^	*U*^23^
Nb1	0.0177 (1)	0.0180 (1)	0.0190 (1)	0.00960 (9)	0.00115 (8)	0.00467 (8)
Nb2	0.0173 (1)	0.0171 (1)	0.0219 (1)	0.01010 (9)	0.00278 (8)	0.00274 (8)
Nb3	0.0150 (1)	0.0168 (1)	0.0208 (1)	0.00477 (8)	−0.00174 (8)	−0.00031 (8)
Nb4	0.0344 (1)	0.0229 (1)	0.0134 (1)	0.0183 (1)	−0.00046 (9)	−0.00156 (8)
Cl1	0.0358 (4)	0.0326 (4)	0.0356 (4)	0.0263 (3)	0.0092 (3)	0.0136 (3)
Cl2	0.0268 (3)	0.0362 (4)	0.0221 (3)	0.0111 (3)	−0.0070 (3)	0.0030 (3)
Cl3	0.0323 (4)	0.0202 (3)	0.0319 (4)	0.0097 (3)	0.0030 (3)	−0.0067 (3)
Cl4	0.0296 (3)	0.0275 (3)	0.0239 (3)	0.0165 (3)	0.0086 (3)	0.0068 (3)
Cl5	0.0156 (3)	0.0345 (4)	0.0377 (4)	0.0118 (3)	0.0001 (3)	0.0010 (3)
Cl6	0.0251 (3)	0.0165 (3)	0.0326 (4)	0.0051 (2)	0.0029 (3)	0.0053 (3)
Cl7	0.0320 (4)	0.0323 (4)	0.0178 (3)	0.0089 (3)	−0.0054 (3)	−0.0070 (3)
Cl8	0.0811 (8)	0.0182 (3)	0.0238 (4)	0.0152 (4)	0.0029 (4)	−0.0004 (3)
S1	0.0696 (8)	0.0401 (5)	0.0467 (6)	0.0325 (5)	−0.0303 (5)	−0.0006 (4)
S2B_a	0.041 (1)	0.091 (2)	0.082 (7)	0.047 (2)	−0.016 (2)	−0.036 (2)
S2A_b	0.036 (2)	0.082 (3)	0.041 (3)	0.041 (2)	0.000 (1)	−0.006 (2)
S3A_c	0.0190 (9)	0.039 (2)	0.053 (3)	0.0002 (9)	−0.005 (1)	0.005 (2)
S3B_d	0.0195 (8)	0.028 (1)	0.047 (3)	0.0042 (8)	0.002 (1)	0.001 (1)
S4	0.093 (1)	0.0522 (7)	0.0262 (5)	0.0316 (7)	−0.0004 (5)	−0.0151 (4)
N1	0.032 (1)	0.033 (1)	0.030 (1)	0.019 (1)	0.006 (1)	0.012 (1)
N2	0.036 (2)	0.032 (1)	0.045 (2)	0.024 (1)	0.016 (1)	0.009 (1)
N3	0.025 (1)	0.032 (2)	0.033 (2)	0.002 (1)	−0.004 (1)	−0.007 (1)
N4	0.088 (3)	0.044 (2)	0.026 (1)	0.049 (2)	−0.003 (2)	−0.006 (1)
N5	0.117 (5)	0.174 (8)	0.042 (3)	0.093 (6)	0.016 (3)	0.030 (4)
N6	0.114 (4)	0.063 (3)	0.033 (2)	0.061 (3)	−0.012 (2)	−0.002 (2)
N7	0.042 (2)	0.042 (2)	0.044 (3)	0.021 (1)	0.00	0.00
N8	0.024 (1)	0.024 (1)	0.040 (3)	0.0118 (6)	0.00	0.00
N9	0.027 (1)	0.027 (1)	0.028 (2)	0.0133 (7)	0.00	0.00
N10	0.051 (2)	0.051 (2)	0.032 (3)	0.026 (1)	0.00	0.00
C1	0.026 (1)	0.026 (1)	0.024 (1)	0.015 (1)	−0.001 (1)	0.001 (1)
C2	0.039 (2)	0.038 (2)	0.067 (3)	0.026 (2)	0.026 (2)	0.015 (2)
C3	0.022 (1)	0.030 (2)	0.043 (2)	0.008 (1)	−0.001 (1)	−0.013 (1)
C4	0.061 (2)	0.025 (2)	0.025 (2)	0.023 (2)	0.005 (2)	0.001 (1)
C5	0.146 (9)	0.17 (1)	0.123 (8)	0.122 (9)	−0.045 (7)	−0.086 (7)
C6	0.19 (1)	0.080 (5)	0.098 (6)	0.099 (7)	−0.065 (6)	−0.047 (4)
C7	0.088 (4)	0.057 (3)	0.058 (3)	0.040 (3)	0.007 (3)	0.001 (3)
C8	0.101 (5)	0.067 (4)	0.040 (3)	0.045 (4)	−0.010 (3)	−0.010 (2)
C9	0.114 (7)	0.25 (1)	0.071 (5)	0.107 (9)	0.015 (5)	0.085 (7)
C10	0.125 (6)	0.062 (4)	0.061 (4)	0.051 (4)	−0.025 (4)	0.007 (3)
C11	0.063 (3)	0.084 (4)	0.060 (3)	0.053 (3)	−0.001 (3)	−0.013 (3)
C12	0.028 (2)	0.032 (2)	0.057 (2)	0.020 (1)	−0.002 (2)	−0.003 (2)
C13	0.026 (2)	0.042 (2)	0.046 (2)	0.015 (2)	−0.002 (2)	−0.002 (2)
C14	0.043 (2)	0.063 (3)	0.043 (2)	0.025 (2)	−0.014 (2)	−0.010 (2)
N11A_e	0.069 (8)	0.038 (5)	0.053 (6)	0.019 (6)	0.014 (5)	0.013 (5)
N12A_e	0.041 (4)	0.038 (4)	0.034 (4)	0.025 (4)	0.012 (3)	0.013 (4)
C15A_e	0.046 (5)	0.059 (6)	0.056 (6)	0.025 (5)	0.013 (4)	0.019 (5)
C16A_e	0.050 (5)	0.065 (6)	0.036 (4)	0.042 (5)	0.011 (4)	0.020 (4)
C17A_e	0.10 (1)	0.065 (7)	0.041 (5)	0.052 (7)	0.024 (6)	0.023 (5)
C18A_e	0.066 (6)	0.070 (7)	0.039 (4)	0.041 (6)	0.020 (4)	0.020 (4)
C19A_e	0.063 (6)	0.074 (7)	0.064 (7)	0.051 (6)	0.009 (5)	−0.001 (6)
C20A_e	0.055 (6)	0.060 (6)	0.060 (6)	0.043 (5)	0.015 (5)	0.010 (5)
N11B_f	0.048 (6)	0.043 (5)	0.043 (5)	0.018 (5)	0.013 (4)	0.012 (5)
N12B_f	0.047 (5)	0.057 (6)	0.043 (5)	0.032 (6)	0.000 (4)	0.001 (5)
C15B_f	0.070 (7)	0.068 (6)	0.035 (4)	0.046 (6)	0.014 (4)	0.012 (4)
C16B_f	0.069 (7)	0.065 (7)	0.053 (6)	0.025 (6)	0.030 (6)	0.010 (5)
C17B_f	0.048 (6)	0.093 (9)	0.060 (6)	0.040 (6)	0.009 (5)	0.021 (6)
C18B_f	0.060 (7)	0.082 (9)	0.12 (1)	0.060 (7)	−0.014 (8)	−0.013 (9)
C19B_f	0.089 (8)	0.099 (9)	0.040 (5)	0.072 (8)	0.021 (5)	0.031 (5)
C20B_f	0.081 (9)	0.10 (1)	0.044 (5)	0.060 (8)	−0.007 (5)	0.009 (6)

### Geometric parameters (Å, º)

**Table:**

Nb1—N1	2.200 (3)	C5—C6	1.48 (1)
Nb1—Cl1	2.4530 (8)	C5—H5A	0.9900
Nb1—Cl6^i^	2.4605 (8)	C5—H5B	0.9900
Nb1—Cl2	2.4617 (8)	C6—H6A	0.9900
Nb1—Cl4^i^	2.4631 (8)	C6—H6B	0.9900
Nb1—Nb2^i^	2.9165 (4)	C7—C8	1.506 (9)
Nb1—Nb3^i^	2.9220 (4)	C7—H7A	0.9900
Nb1—Nb2	2.9221 (4)	C7—H7B	0.9900
Nb1—Nb3	2.9241 (4)	C8—H8A	0.9900
Nb2—N2	2.211 (3)	C8—H8B	0.9900
Nb2—Cl1	2.4467 (8)	C9—C10	1.51 (1)
Nb2—Cl4	2.4527 (8)	C9—H9A	0.9900
Nb2—Cl3	2.4593 (8)	C9—H9B	0.9900
Nb2—Cl5	2.4651 (8)	C10—H10A	0.9900
Nb2—Nb1^i^	2.9164 (4)	C10—H10B	0.9900
Nb2—Nb3^i^	2.9207 (4)	C11—C12	1.534 (7)
Nb2—Nb3	2.9293 (4)	C11—H11A	0.9900
Nb3—N3	2.201 (3)	C11—H11B	0.9900
Nb3—Cl2	2.4561 (8)	C12—H12A	0.9900
Nb3—Cl5	2.4573 (8)	C12—H12B	0.9900
Nb3—Cl3^i^	2.4602 (8)	C13—C14	1.532 (6)
Nb3—Cl6	2.4627 (8)	C13—H13A	0.9900
Nb3—Nb2^i^	2.9207 (4)	C13—H13B	0.9900
Nb3—Nb1^i^	2.9221 (4)	C14—H14A	0.9900
Nb4—N4	2.199 (3)	C14—H14B	0.9900
Nb4—Cl8	2.456 (1)	N11A_e—C19A_e	1.45 (2)
Nb4—Cl7^ii^	2.4568 (9)	N11A_e—C17A_e	1.47 (2)
Nb4—Cl7	2.4587 (9)	N11A_e—C15A_e	1.50 (2)
Nb4—Cl8^iii^	2.462 (1)	N12A_e—C20A_e	1.47 (1)
Nb4—Nb4^iii^	2.9199 (5)	N12A_e—C16A_e	1.51 (2)
Nb4—Nb4^iv^	2.9199 (5)	N12A_e—C18A_e	1.51 (1)
Nb4—Nb4^ii^	2.9213 (5)	N12A_e—H12N_e	1.0000
Nb4—Nb4^v^	2.9214 (5)	C15A_e—C16A_e	1.54 (2)
Cl3—Nb3^i^	2.4602 (8)	C15A_e—H15A_e	0.9900
Cl4—Nb1^i^	2.4629 (8)	C15A_e—H15B_e	0.9900
Cl6—Nb1^i^	2.4606 (8)	C16A_e—H16A_e	0.9900
Cl7—Nb4^v^	2.4569 (9)	C16A_e—H16B_e	0.9900
Cl8—Nb4^iv^	2.462 (1)	C17A_e—C18A_e	1.50 (2)
S1—C1	1.612 (3)	C17A_e—H17A_e	0.9900
S2B_a—C2	1.557 (5)	C17A_e—H17B_e	0.9900
S2A_b—C2	1.692 (7)	C18A_e—H18A_e	0.9900
S3A_c—C3	1.613 (4)	C18A_e—H18B_e	0.9900
S3B_d—C3	1.646 (5)	C19A_e—C20A_e	1.53 (2)
S4—C4	1.611 (4)	C19A_e—H19A_e	0.9900
N1—C1	1.149 (4)	C19A_e—H19B_e	0.9900
N2—C2	1.171 (5)	C20A_e—H20A_e	0.9900
N3—C3	1.156 (4)	C20A_e—H20B_e	0.9900
N4—C4	1.158 (5)	N11B_f—C17B_f	1.45 (2)
N5—C7	1.471 (8)	N11B_f—C15B_f	1.51 (1)
N5—C9	1.472 (9)	N11B_f—C19B_f	1.52 (2)
N5—C5	1.49 (1)	N11B_f—H11N_f	1.0000
N5—H5N	1.0000	N12B_f—C18B_f	1.43 (2)
N6—C6	1.466 (9)	N12B_f—C20B_f	1.48 (2)
N6—C10	1.495 (7)	N12B_f—C16B_f	1.48 (2)
N6—C8	1.512 (7)	C15B_f—C16B_f	1.53 (2)
N7—C11	1.479 (6)	C15B_f—H15C_f	0.9900
N7—C11^vi^	1.479 (6)	C15B_f—H15D_f	0.9900
N7—C11^vii^	1.480 (6)	C16B_f—H16C_f	0.9900
N8—C12^vii^	1.488 (4)	C16B_f—H16D_f	0.9900
N8—C12^vi^	1.488 (4)	C17B_f—C18B_f	1.52 (2)
N8—C12	1.489 (4)	C17B_f—H17C_f	0.9900
N8—H8N	1.0000	C17B_f—H17D_f	0.9900
N9—C13	1.483 (4)	C18B_f—H18C_f	0.9900
N9—C13^vii^	1.483 (4)	C18B_f—H18D_f	0.9900
N9—C13^vi^	1.483 (4)	C19B_f—C20B_f	1.51 (2)
N10—C14	1.489 (5)	C19B_f—H19C_f	0.9900
N10—C14^vii^	1.489 (5)	C19B_f—H19D_f	0.9900
N10—C14^vi^	1.489 (5)	C20B_f—H20C_f	0.9900
N10—H10N	1.0000	C20B_f—H20D_f	0.9900
			
N1—Nb1—Cl1	80.10 (8)	C12^vi^—N8—C12	108.6 (3)
N1—Nb1—Cl6^i^	82.43 (9)	C12^vii^—N8—H8N	110.3
Cl1—Nb1—Cl6^i^	87.98 (3)	C12^vi^—N8—H8N	110.3
N1—Nb1—Cl2	80.31 (9)	C12—N8—H8N	110.3
Cl1—Nb1—Cl2	89.84 (3)	C13—N9—C13^vii^	109.0 (3)
Cl6^i^—Nb1—Cl2	162.72 (3)	C13—N9—C13^vi^	109.0 (3)
N1—Nb1—Cl4^i^	83.16 (8)	C13^vii^—N9—C13^vi^	109.0 (3)
Cl1—Nb1—Cl4^i^	163.08 (3)	C14—N10—C14^vii^	109.3 (3)
Cl6^i^—Nb1—Cl4^i^	87.38 (3)	C14—N10—C14^vi^	109.3 (3)
Cl2—Nb1—Cl4^i^	89.77 (3)	C14^vii^—N10—C14^vi^	109.3 (3)
N1—Nb1—Nb2^i^	136.57 (8)	C14—N10—H10N	109.6
Cl1—Nb1—Nb2^i^	143.33 (2)	C14^vii^—N10—H10N	109.6
Cl6^i^—Nb1—Nb2^i^	95.69 (2)	C14^vi^—N10—H10N	109.6
Cl2—Nb1—Nb2^i^	96.27 (2)	N1—C1—S1	179.1 (3)
Cl4^i^—Nb1—Nb2^i^	53.44 (2)	N2—C2—S2B_a	166.7 (8)
N1—Nb1—Nb3^i^	136.00 (9)	N2—C2—S2A_b	172.2 (6)
Cl1—Nb1—Nb3^i^	94.66 (2)	N3—C3—S3A_c	169.8 (7)
Cl6^i^—Nb1—Nb3^i^	53.63 (2)	N3—C3—S3B_d	169.7 (6)
Cl2—Nb1—Nb3^i^	143.65 (2)	N4—C4—S4	179.2 (4)
Cl4^i^—Nb1—Nb3^i^	95.66 (2)	C6—C5—N5	103.6 (7)
Nb2^i^—Nb1—Nb3^i^	60.229 (9)	C6—C5—H5A	111.0
N1—Nb1—Nb2	133.33 (8)	N5—C5—H5A	111.0
Cl1—Nb1—Nb2	53.29 (2)	C6—C5—H5B	111.0
Cl6^i^—Nb1—Nb2	96.66 (2)	N5—C5—H5B	111.0
Cl2—Nb1—Nb2	95.74 (2)	H5A—C5—H5B	109.0
Cl4^i^—Nb1—Nb2	143.51 (2)	N6—C6—C5	114.6 (6)
Nb2^i^—Nb1—Nb2	90.08 (1)	N6—C6—H6A	108.6
Nb3^i^—Nb1—Nb2	59.97 (1)	C5—C6—H6A	108.6
N1—Nb1—Nb3	133.73 (9)	N6—C6—H6B	108.6
Cl1—Nb1—Nb3	96.97 (2)	C5—C6—H6B	108.6
Cl6^i^—Nb1—Nb3	143.84 (2)	H6A—C6—H6B	107.6
Cl2—Nb1—Nb3	53.43 (2)	N5—C7—C8	106.2 (6)
Cl4^i^—Nb1—Nb3	96.37 (2)	N5—C7—H7A	110.5
Nb2^i^—Nb1—Nb3	60.009 (8)	C8—C7—H7A	110.5
Nb3^i^—Nb1—Nb3	90.22 (1)	N5—C7—H7B	110.5
Nb2—Nb1—Nb3	60.140 (9)	C8—C7—H7B	110.5
N2—Nb2—Cl1	80.88 (9)	H7A—C7—H7B	108.7
N2—Nb2—Cl4	82.00 (9)	C7—C8—N6	110.8 (5)
Cl1—Nb2—Cl4	162.82 (3)	C7—C8—H8A	109.5
N2—Nb2—Cl3	83.5 (1)	N6—C8—H8A	109.5
Cl1—Nb2—Cl3	88.20 (3)	C7—C8—H8B	109.5
Cl4—Nb2—Cl3	88.42 (3)	N6—C8—H8B	109.5
N2—Nb2—Cl5	79.4 (1)	H8A—C8—H8B	108.1
Cl1—Nb2—Cl5	89.22 (3)	N5—C9—C10	106.3 (6)
Cl4—Nb2—Cl5	89.08 (3)	N5—C9—H9A	110.5
Cl3—Nb2—Cl5	162.92 (3)	C10—C9—H9A	110.5
N2—Nb2—Nb1^i^	135.74 (8)	N5—C9—H9B	110.5
Cl1—Nb2—Nb1^i^	143.38 (2)	C10—C9—H9B	110.5
Cl4—Nb2—Nb1^i^	53.77 (2)	H9A—C9—H9B	108.7
Cl3—Nb2—Nb1^i^	95.52 (2)	N6—C10—C9	110.2 (6)
Cl5—Nb2—Nb1^i^	96.61 (2)	N6—C10—H10A	109.6
N2—Nb2—Nb3^i^	137.10 (9)	C9—C10—H10A	109.6
Cl1—Nb2—Nb3^i^	94.83 (2)	N6—C10—H10B	109.6
Cl4—Nb2—Nb3^i^	96.68 (2)	C9—C10—H10B	109.6
Cl3—Nb2—Nb3^i^	53.59 (2)	H10A—C10—H10B	108.1
Cl5—Nb2—Nb3^i^	143.48 (2)	N7—C11—C12	109.6 (4)
Nb1^i^—Nb2—Nb3^i^	60.125 (8)	N7—C11—H11A	109.7
N2—Nb2—Nb1	134.24 (8)	C12—C11—H11A	109.7
Cl1—Nb2—Nb1	53.49 (2)	N7—C11—H11B	109.7
Cl4—Nb2—Nb1	143.69 (2)	C12—C11—H11B	109.7
Cl3—Nb2—Nb1	96.83 (2)	H11A—C11—H11B	108.2
Cl5—Nb2—Nb1	95.17 (2)	N8—C12—C11	109.0 (4)
Nb1^i^—Nb2—Nb1	89.93 (1)	N8—C12—H12A	109.9
Nb3^i^—Nb2—Nb1	60.014 (9)	C11—C12—H12A	109.9
N2—Nb2—Nb3	132.7 (1)	N8—C12—H12B	109.9
Cl1—Nb2—Nb3	96.98 (2)	C11—C12—H12B	109.9
Cl4—Nb2—Nb3	95.70 (2)	H12A—C12—H12B	108.3
Cl3—Nb2—Nb3	143.72 (2)	N9—C13—C14	109.1 (4)
Cl5—Nb2—Nb3	53.36 (2)	N9—C13—H13A	109.9
Nb1^i^—Nb2—Nb3	59.983 (9)	C14—C13—H13A	109.9
Nb3^i^—Nb2—Nb3	90.147 (9)	N9—C13—H13B	109.9
Nb1—Nb2—Nb3	59.963 (9)	C14—C13—H13B	109.9
N3—Nb3—Cl2	83.07 (9)	H13A—C13—H13B	108.3
N3—Nb3—Cl5	80.0 (1)	N10—C14—C13	108.1 (4)
Cl2—Nb3—Cl5	87.27 (3)	N10—C14—H14A	110.1
N3—Nb3—Cl3^i^	83.1 (1)	C13—C14—H14A	110.1
Cl2—Nb3—Cl3^i^	88.24 (3)	N10—C14—H14B	110.1
Cl5—Nb3—Cl3^i^	162.87 (3)	C13—C14—H14B	110.1
N3—Nb3—Cl6	79.96 (9)	H14A—C14—H14B	108.4
Cl2—Nb3—Cl6	163.01 (3)	C19A_e—N11A_e—C17A_e	110 (1)
Cl5—Nb3—Cl6	88.87 (3)	C19A_e—N11A_e—C15A_e	110.9 (9)
Cl3^i^—Nb3—Cl6	90.63 (3)	C17A_e—N11A_e—C15A_e	108 (1)
N3—Nb3—Nb2^i^	136.56 (9)	C20A_e—N12A_e—C16A_e	108.6 (9)
Cl2—Nb3—Nb2^i^	96.29 (2)	C20A_e—N12A_e—C18A_e	109.1 (8)
Cl5—Nb3—Nb2^i^	143.44 (2)	C16A_e—N12A_e—C18A_e	107.0 (9)
Cl3^i^—Nb3—Nb2^i^	53.56 (2)	C20A_e—N12A_e—H12N_e	110.7
Cl6—Nb3—Nb2^i^	96.65 (2)	C16A_e—N12A_e—H12N_e	110.7
N3—Nb3—Nb1^i^	133.51 (9)	C18A_e—N12A_e—H12N_e	110.7
Cl2—Nb3—Nb1^i^	143.38 (2)	N11A_e—C15A_e—C16A_e	108.2 (9)
Cl5—Nb3—Nb1^i^	96.64 (2)	N11A_e—C15A_e—H15A_e	110.1
Cl3^i^—Nb3—Nb1^i^	96.81 (2)	C16A_e—C15A_e—H15A_e	110.1
Cl6—Nb3—Nb1^i^	53.56 (2)	N11A_e—C15A_e—H15B_e	110.1
Nb2^i^—Nb3—Nb1^i^	60.019 (9)	C16A_e—C15A_e—H15B_e	110.1
N3—Nb3—Nb1	136.67 (9)	H15A_e—C15A_e—H15B_e	108.4
Cl2—Nb3—Nb1	53.60 (2)	N12A_e—C16A_e—C15A_e	110.5 (7)
Cl5—Nb3—Nb1	95.30 (2)	N12A_e—C16A_e—H16A_e	109.5
Cl3^i^—Nb3—Nb1	95.31 (2)	C15A_e—C16A_e—H16A_e	109.5
Cl6—Nb3—Nb1	143.32 (2)	N12A_e—C16A_e—H16B_e	109.5
Nb2^i^—Nb3—Nb1	59.867 (8)	C15A_e—C16A_e—H16B_e	109.5
Nb1^i^—Nb3—Nb1	89.78 (1)	H16A_e—C16A_e—H16B_e	108.1
N3—Nb3—Nb2	133.54 (9)	N11A_e—C17A_e—C18A_e	110.4 (9)
Cl2—Nb3—Nb2	95.68 (2)	N11A_e—C17A_e—H17A_e	109.6
Cl5—Nb3—Nb2	53.60 (2)	C18A_e—C17A_e—H17A_e	109.6
Cl3^i^—Nb3—Nb2	143.40 (2)	N11A_e—C17A_e—H17B_e	109.6
Cl6—Nb3—Nb2	95.31 (2)	C18A_e—C17A_e—H17B_e	109.6
Nb2^i^—Nb3—Nb2	89.855 (9)	H17A_e—C17A_e—H17B_e	108.1
Nb1^i^—Nb3—Nb2	59.790 (9)	C17A_e—C18A_e—N12A_e	110.1 (8)
Nb1—Nb3—Nb2	59.898 (9)	C17A_e—C18A_e—H18A_e	109.6
N4—Nb4—Cl8	82.8 (1)	N12A_e—C18A_e—H18A_e	109.6
N4—Nb4—Cl7^ii^	80.0 (1)	C17A_e—C18A_e—H18B_e	109.6
Cl8—Nb4—Cl7^ii^	162.74 (3)	N12A_e—C18A_e—H18B_e	109.6
N4—Nb4—Cl7	79.5 (1)	H18A_e—C18A_e—H18B_e	108.2
Cl8—Nb4—Cl7	90.39 (4)	N11A_e—C19A_e—C20A_e	109.7 (8)
Cl7^ii^—Nb4—Cl7	88.07 (4)	N11A_e—C19A_e—H19A_e	109.7
N4—Nb4—Cl8^iii^	83.4 (1)	C20A_e—C19A_e—H19A_e	109.7
Cl8—Nb4—Cl8^iii^	88.75 (1)	N11A_e—C19A_e—H19B_e	109.7
Cl7^ii^—Nb4—Cl8^iii^	87.71 (4)	C20A_e—C19A_e—H19B_e	109.7
Cl7—Nb4—Cl8^iii^	162.93 (3)	H19A_e—C19A_e—H19B_e	108.2
N4—Nb4—Nb4^iii^	136.9 (1)	N12A_e—C20A_e—C19A_e	110.7 (9)
Cl8—Nb4—Nb4^iii^	95.17 (3)	N12A_e—C20A_e—H20A_e	109.5
Cl7^ii^—Nb4—Nb4^iii^	96.27 (2)	C19A_e—C20A_e—H20A_e	109.5
Cl7—Nb4—Nb4^iii^	143.51 (2)	N12A_e—C20A_e—H20B_e	109.5
Cl8^iii^—Nb4—Nb4^iii^	53.49 (2)	C19A_e—C20A_e—H20B_e	109.5
N4—Nb4—Nb4^iv^	136.4 (1)	H20A_e—C20A_e—H20B_e	108.1
Cl8—Nb4—Nb4^iv^	53.67 (2)	C17B_f—N11B_f—C15B_f	108.8 (9)
Cl7^ii^—Nb4—Nb4^iv^	143.57 (2)	C17B_f—N11B_f—C19B_f	110 (1)
Cl7—Nb4—Nb4^iv^	96.22 (2)	C15B_f—N11B_f—C19B_f	106 (1)
Cl8^iii^—Nb4—Nb4^iv^	96.98 (3)	C17B_f—N11B_f—H11N_f	110.6
Nb4^iii^—Nb4—Nb4^iv^	60.03 (1)	C15B_f—N11B_f—H11N_f	110.6
N4—Nb4—Nb4^ii^	133.5 (1)	C19B_f—N11B_f—H11N_f	110.6
Cl8—Nb4—Nb4^ii^	143.64 (2)	C18B_f—N12B_f—C20B_f	109 (1)
Cl7^ii^—Nb4—Nb4^ii^	53.57 (2)	C18B_f—N12B_f—C16B_f	109 (1)
Cl7—Nb4—Nb4^ii^	95.77 (2)	C20B_f—N12B_f—C16B_f	107 (1)
Cl8^iii^—Nb4—Nb4^ii^	95.01 (3)	N11B_f—C15B_f—C16B_f	108.4 (8)
Nb4^iii^—Nb4—Nb4^ii^	59.987 (6)	N11B_f—C15B_f—H15C_f	110.0
Nb4^iv^—Nb4—Nb4^ii^	90.0	C16B_f—C15B_f—H15C_f	110.0
N4—Nb4—Nb4^v^	133.0 (1)	N11B_f—C15B_f—H15D_f	110.0
Cl8—Nb4—Nb4^v^	97.08 (3)	C16B_f—C15B_f—H15D_f	110.0
Cl7^ii^—Nb4—Nb4^v^	95.81 (2)	H15C_f—C15B_f—H15D_f	108.4
Cl7—Nb4—Nb4^v^	53.51 (2)	N12B_f—C16B_f—C15B_f	110.4 (9)
Cl8^iii^—Nb4—Nb4^v^	143.46 (2)	N12B_f—C16B_f—H16C_f	109.6
Nb4^iii^—Nb4—Nb4^v^	90.0	C15B_f—C16B_f—H16C_f	109.6
Nb4^iv^—Nb4—Nb4^v^	59.986 (6)	N12B_f—C16B_f—H16D_f	109.6
Nb4^ii^—Nb4—Nb4^v^	60.0	C15B_f—C16B_f—H16D_f	109.6
Nb2—Cl1—Nb1	73.22 (2)	H16C_f—C16B_f—H16D_f	108.1
Nb3—Cl2—Nb1	72.97 (2)	N11B_f—C17B_f—C18B_f	108.6 (9)
Nb2—Cl3—Nb3^i^	72.84 (2)	N11B_f—C17B_f—H17C_f	110.0
Nb2—Cl4—Nb1^i^	72.78 (2)	C18B_f—C17B_f—H17C_f	110.0
Nb3—Cl5—Nb2	73.04 (2)	N11B_f—C17B_f—H17D_f	110.0
Nb1^i^—Cl6—Nb3	72.81 (2)	C18B_f—C17B_f—H17D_f	110.0
Nb4^v^—Cl7—Nb4	72.93 (2)	H17C_f—C17B_f—H17D_f	108.4
Nb4—Cl8—Nb4^iv^	72.84 (3)	N12B_f—C18B_f—C17B_f	112.4 (9)
C1—N1—Nb1	158.1 (3)	N12B_f—C18B_f—H18C_f	109.1
C2—N2—Nb2	143.3 (4)	C17B_f—C18B_f—H18C_f	109.1
C3—N3—Nb3	154.5 (3)	N12B_f—C18B_f—H18D_f	109.1
C4—N4—Nb4	146.7 (3)	C17B_f—C18B_f—H18D_f	109.1
C7—N5—C9	110.4 (7)	H18C_f—C18B_f—H18D_f	107.9
C7—N5—C5	109.4 (6)	C20B_f—C19B_f—N11B_f	109.0 (8)
C9—N5—C5	112.4 (9)	C20B_f—C19B_f—H19C_f	109.9
C7—N5—H5N	108.1	N11B_f—C19B_f—H19C_f	109.9
C9—N5—H5N	108.1	C20B_f—C19B_f—H19D_f	109.9
C5—N5—H5N	108.1	N11B_f—C19B_f—H19D_f	109.9
C6—N6—C10	107.9 (6)	H19C_f—C19B_f—H19D_f	108.3
C6—N6—C8	105.2 (5)	N12B_f—C20B_f—C19B_f	110 (1)
C10—N6—C8	105.1 (5)	N12B_f—C20B_f—H20C_f	109.6
C11—N7—C11^vi^	108.6 (3)	C19B_f—C20B_f—H20C_f	109.6
C11—N7—C11^vii^	108.6 (3)	N12B_f—C20B_f—H20D_f	109.6
C11^vi^—N7—C11^vii^	108.6 (3)	C19B_f—C20B_f—H20D_f	109.6
C12^vii^—N8—C12^vi^	108.6 (3)	H20C_f—C20B_f—H20D_f	108.1
C12^vii^—N8—C12	108.6 (3)		
			
Nb2—N2—C2—S2B_a	−156 (2)	C17A_e—N11A_e—C15A_e—C16A_e	−63 (1)
Nb3—N3—C3—S3A_c	−130 (2)	C20A_e—N12A_e—C16A_e—C15A_e	−60 (1)
Nb3—N3—C3—S3B_d	51 (3)	C18A_e—N12A_e—C16A_e—C15A_e	57 (1)
C7—N5—C5—C6	−75.1 (9)	N11A_e—C15A_e—C16A_e—N12A_e	3 (1)
C9—N5—C5—C6	48.0 (9)	C19A_e—N11A_e—C17A_e—C18A_e	−61 (1)
C10—N6—C6—C5	−68.9 (9)	C15A_e—N11A_e—C17A_e—C18A_e	60 (1)
C8—N6—C6—C5	42.8 (9)	N11A_e—C17A_e—C18A_e—N12A_e	3 (2)
N5—C5—C6—N6	22 (1)	C20A_e—N12A_e—C18A_e—C17A_e	56 (1)
C9—N5—C7—C8	−73.2 (9)	C16A_e—N12A_e—C18A_e—C17A_e	−61 (1)
C5—N5—C7—C8	51.2 (8)	C17A_e—N11A_e—C19A_e—C20A_e	58 (1)
N5—C7—C8—N6	19.7 (7)	C15A_e—N11A_e—C19A_e—C20A_e	−61 (1)
C6—N6—C8—C7	−67.5 (7)	C16A_e—N12A_e—C20A_e—C19A_e	58 (1)
C10—N6—C8—C7	46.2 (7)	C18A_e—N12A_e—C20A_e—C19A_e	−58 (1)
C7—N5—C9—C10	49 (1)	N11A_e—C19A_e—C20A_e—N12A_e	2 (1)
C5—N5—C9—C10	−73 (1)	C17B_f—N11B_f—C15B_f—C16B_f	67 (1)
C6—N6—C10—C9	40.7 (9)	C19B_f—N11B_f—C15B_f—C16B_f	−51 (1)
C8—N6—C10—C9	−71.2 (8)	C18B_f—N12B_f—C16B_f—C15B_f	−49 (2)
N5—C9—C10—N6	22 (1)	C20B_f—N12B_f—C16B_f—C15B_f	69 (1)
C11^vi^—N7—C11—C12	−49.6 (5)	N11B_f—C15B_f—C16B_f—N12B_f	−14 (2)
C11^vii^—N7—C11—C12	68.4 (5)	C15B_f—N11B_f—C17B_f—C18B_f	−52 (1)
C12^vii^—N8—C12—C11	−49.6 (5)	C19B_f—N11B_f—C17B_f—C18B_f	64 (1)
C12^vi^—N8—C12—C11	68.3 (4)	C20B_f—N12B_f—C18B_f—C17B_f	−51 (2)
N7—C11—C12—N8	−16.1 (5)	C16B_f—N12B_f—C18B_f—C17B_f	65 (2)
C13^vii^—N9—C13—C14	69.6 (4)	N11B_f—C17B_f—C18B_f—N12B_f	−12 (2)
C13^vi^—N9—C13—C14	−49.2 (4)	C17B_f—N11B_f—C19B_f—C20B_f	−49 (1)
C14^vii^—N10—C14—C13	−49.7 (5)	C15B_f—N11B_f—C19B_f—C20B_f	68 (1)
C14^vi^—N10—C14—C13	69.9 (4)	C18B_f—N12B_f—C20B_f—C19B_f	66 (1)
N9—C13—C14—N10	−17.3 (5)	C16B_f—N12B_f—C20B_f—C19B_f	−51 (1)
C19A_e—N11A_e—C15A_e—C16A_e	58 (1)	N11B_f—C19B_f—C20B_f—N12B_f	−15 (1)

Symmetry codes: (i) −*x*+1/3, −*y*+2/3, −*z*+2/3; (ii) −*y*+1, *x*−*y*, *z*; (iii) *x*−*y*+1/3, *x*−1/3, −*z*+2/3; (iv) *y*+1/3, −*x*+*y*+2/3, −*z*+2/3; (v) −*x*+*y*+1, −*x*+1, *z*; (vi) −*x*+*y*, −*x*+1, *z*; (vii) −*y*+1, *x*−*y*+1, *z*.

### Hydrogen-bond geometry (Å, º)

**Table:**

*D*—H···*A*	*D*—H	H···*A*	*D*···*A*	*D*—H···*A*
N5—H5*N*···Cl5	1.00	2.46	3.368 (7)	150
N8—H8*N*···N9	1.00	1.80	2.795 (8)	180
N10—H10*N*···Cl7^viii^	1.00	2.90	3.695 (5)	137
N12A—H12N···N6^iii^	1.00	1.59	2.59 (1)	176
N10—H10*N*···Cl7^ix^	1.00	2.90	3.695 (5)	137
N10—H10*N*···Cl7^x^	1.00	2.90	3.695 (5)	137

Symmetry codes: (iii) *x*−*y*+1/3, *x*−1/3, −*z*+2/3; (viii) −*x*+1, −*y*+1, −*z*+1; (ix) *y*, −*x*+*y*+1, −*z*+1; (x) *x*−*y*, *x*, −*z*+1.
